# Combining Topical Oxygen and Negative-Pressure Wound Therapy: New Insights from a Pilot Study on Chronic Wound Treatment

**DOI:** 10.3390/jcm14155564

**Published:** 2025-08-07

**Authors:** Bartosz Molasy, Mateusz Frydrych, Rafał Kuchciński, Stanisław Głuszek

**Affiliations:** 1 Medical College, The Jan Kochanowski University, 25-317 Kielce, Poland; 2 Department of General Surgery, St. Alexander Hospital, 25-316 Kielce, Poland; frydrychmf@gmail.com (M.F.); rafalk007@tlen.pl (R.K.); 3 Department of Surgical Oncology, Holy Cross Cancer Center, 25-734 Kielce, Poland; sgluszek@wp.pl; 4 Institute of Genetics and Animal Biotechnology, Polish Academy of Sciences, 05-552 Magdalenka, Poland

**Keywords:** chronic wounds, wound healing, combined therapy, topical oxygen therapy, negative-pressure wound therapy, biomarkers, thermography

## Abstract

**Background:** Chronic wounds are a growing clinical challenge due to their prolonged healing time and associated healthcare burden. Combined therapeutic approaches, including topical oxygen therapy (TOT) and negative-pressure wound therapy (NPWT), have shown promise in enhancing wound healing. This pilot exploratory study aimed to assess the clinical effectiveness of combined TOT and NPWT in chronic wound treatment and to explore the prognostic value of selected laboratory and thermographic markers. **Methods:** Eighteen patients with chronic wounds due to type 2 diabetes mellitus or chronic venous insufficiency were treated with either TOT alone (control group) or TOT combined with NPWT (intervention group). Wound characteristics, thermographic data, and laboratory parameters (NLR, MLR, PLR, CRP, and total protein) were collected at baseline and during therapy. The primary endpoints were the total treatment duration and complete wound closure. Statistical analyses were exploratory and used non-parametric tests, correlation analyses, and simple linear regression. **Results:** Ulcer duration was significantly associated with the wound surface area. Lower serum total protein levels correlated negatively with ulcer duration, wound size, and granulation tissue area. A significant reduction in treatment duration was observed in the intervention group compared to the controls. One strong correlation was found between MLR and peripheral wound temperature on day 7 in the control group. No significant group differences were observed in wound size or thermographic measures after one week of treatment. **Conclusions:** Combining TOT and NPWT may reduce treatment duration in chronic wound management. Selected laboratory and thermographic markers show promise as prognostic tools. These exploratory findings require confirmation in larger, randomized trials.

## 1. Introduction

Chronic wounds, typically defined as wounds that fail to heal within six weeks, remain a major clinical and socioeconomic burden and markedly diminish patients’ quality of life [1,2]. Epidemiological studies estimate that chronic wounds affect approximately 1–2% of the population in developed countries, with elderly individuals and patients with diabetes or chronic venous insufficiency being particularly at risk [3,4]. The management of these wounds generates high healthcare costs and underscores the need for effective, personalized treatment strategies [3,4]. Among advanced treatment modalities, negative-pressure wound therapy (NPWT) and topical oxygen therapy (TOT) are two of the most frequently used yet mechanistically distinct approaches.

NPWT applies sub-atmospheric pressure across a sealed foam interface, promoting granulation tissue formation, controlling exudate, and reducing edema [5]. However, perfusion measurements indicate that the immediate increase in blood flow may be limited by the mechanical action of negative pressure on blood vessels, with improved vascularization occurring gradually through angiogenesis [6]. Conversely, TOT delivers high-concentration oxygen directly to the wound bed, supporting fibroblast proliferation, collagen deposition, and bacterial killing [7]. TOT techniques vary, including continuous or cyclic oxygen delivery, often in a humidified environment, which optimizes the metabolic and enzymatic processes essential for healing [8]. Because oxygen tension is a limiting factor even under NPWT dressings, there is growing interest in combining both approaches to exploit their complementary mechanisms.

Evidence for this synergy is accumulating. Recent studies, including randomized controlled trials and meta-analyses, suggest that combining NPWT with adjunctive oxygen therapy significantly improves healing outcomes compared to monotherapy, particularly in complex or refractory wounds. A single-center randomized controlled trial on stage IV sacrococcygeal pressure ulcers reported that adding local oxygen to vacuum sealing drainage reduced the median healing time by 30 days compared to NPWT alone [9]. A 2024 systematic review and meta-analysis pooled ten studies and showed that adjunctive oxygen—whether topical or hyperbaric—combined with NPWT improved the odds of complete closure by 1.8-fold versus NPWT monotherapy [10].

Equally important is the ability to stratify patients who are most likely to benefit from this treatment. Readily available inflammatory ratios—neutrophil-to-lymphocyte (NLR), monocyte-to-lymphocyte (MLR), and platelet-to-lymphocyte (PLR)—have demonstrated prognostic value in diabetic and venous ulcers [11,12]. Serum total protein serves as a low-cost surrogate of nutritional reserve, deficiencies of which can delay collagen synthesis and epithelialization [13,14]. Infra-red thermography offers a non-contact method of monitoring local perfusion; however, its predictive utility in chronic-wound trajectories is not firmly established [15].

In this context, the present pilot study prospectively compares combined TOT + NPWT with TOT alone in a clinically homogeneous cohort and explores whether baseline laboratory markers or early thermographic patterns can refine prognostic assessment. The overarching aim is to generate feasibility data that will inform personalized treatment algorithms for hard-to-heal wounds.

## 2. Materials and Methods

### 2.1. Study Design and Setting

This prospective, non-randomized pilot study was conducted between 1 January and 31 December 2024 to assess the feasibility and preliminary clinical impact of combining topical oxygen therapy (TOT) with negative-pressure wound therapy (NPWT) in patients with chronic lower limb ulcers. The primary objective was to generate pilot data to inform the design of future randomized controlled trials. The study received institutional ethics approval (Bioethics Committee no. 1/2024), and all participants provided written informed consent in accordance with the Declaration of Helsinki.

### 2.2. Study Population and Eligibility Criteria

Adults aged ≥18 years with a chronic lower-limb ulcer of ≥6 weeks duration caused by type 2 diabetes mellitus or chronic venous insufficiency were eligible. Additional inclusion criteria included wound surface areas < 200 cm^2^. Exclusion criteria comprised a lack of informed consent, critical limb ischemia (defined as an absence of distal arterial flow on duplex ultrasound or CT angiography), severe anemia (hemoglobin < 8 g/dL), advanced renal failure (eGFR < 30 mL/min/1.73 m^2^), active malignancy, a body mass index < 18.5 kg/m^2^, immunosuppressive therapy, or necrotizing soft tissue infection.

### 2.3. Allocation and Interventions

Participants were allocated using a 1:1 ratio to receive either TOT treatment alone (control group) or the combined TOT + NPWT treatment (intervention group) using a computer-generated random list (Excel RAND). Allocation was open-label and not concealed. TOT was administered on days 1–4 using a single-use oxygen delivery chamber (O_2_Boot™, GWR Medical, Inc., Chadds Ford, PA, USA) for 1.5 h per day at a continuous flow rate of 10 L/min, consistent with 2022 Delphi consensus recommendations [16]. In the intervention group, NPWT (Vivano^®^ Tec, Hartmann, Germany) was applied from days 5 to 7 at −125 mmHg continuous pressure, with dressing changes every 48 h in accordance with international guidelines [17].

Both groups received identical adjunctive care, including surgical debridement at each visit, irrigation with 0.05% sodium hypochlorite, and compression therapy for venous ulcers. Compression was delivered using class II medical compression stockings (≈40 mmHg at the ankle). All interventions were administered by the same three-person wound-care team.

### 2.4. Outcome Measures

Primary endpoints were as follows: (i) total treatment duration (defined as weeks from enrollment to complete epithelialization confirmed clinically) and (ii) the proportion of wounds achieving complete closure within the 9-month follow-up period. Secondary outcomes included early changes in wound morphology and temperature, as well as associations between laboratory parameters and wound-healing dynamics.

Wound dimensions (area, granulation, necrosis) were quantified at baseline, day 4, and day 7 using a validated three-dimensional imaging system (WoundMatrix™ (WoundMatrix, Inc., Chadds Ford, PA, USA)). Infrared thermographic data were acquired using an FLIR-One Pro camera (Teledyne FLIR LLC, Wilsonville, OR, USA) under controlled ambient conditions (22 ± 1 °C; 50% humidity), with measurements taken over the wound bed and surrounding a 1–2 cm peri-wound zone. Baseline venous blood samples were analyzed for complete blood count, C-reactive protein (CRP), and serum total protein. NLR, MLR, and PLR were calculated accordingly.

### 2.5. Statistical Analysis

Descriptive statistics included the median (interquartile range, IQR) for continuous variables and frequency (percentage) for categorical data. Between-group comparisons were made using the Mann–Whitney U test and Fisher’s exact test. Associations between variables were assessed using Spearman’s rank correlation and simple linear regression. Due to the exploratory nature of the study and small sample size, no multivariable analysis or correction for multiple comparisons was performed.

Statistical analyses were performed using R version 4.3.1 (R Foundation for Statistical Computing, Vienna, Austria). A post hoc power analysis was conducted using G*Power version 3.1 (Heinrich Heine University Düsseldorf, Düsseldorf, Germany); based on the observed between-group difference in treatment duration (Δ = 24 weeks), the study had an estimated power of 78% to detect a statistically significant difference at a two-sided alpha level of 0.05.

## 3. Results

A total of 18 patients were included in the analysis. Eight patients were men and ten were women. The intervention group included nine patients who received topical oxygen therapy combined with negative-pressure wound therapy (NPWT), and the control group included nine patients treated with topical oxygen therapy alone.

In 16 cases, the chronic wound was associated with venous insufficiency, while in two cases, the etiology was diabetic. Baseline characteristics were comparable between groups, with no statistically significant differences in age, sex, ulcer duration, wound area, or serum total protein (Table 1).

At baseline, the median granulation tissue area was 9.2 cm^2^ (IQR: 6.1–14.5) in the TOT + NPWT group and 6.3 cm^2^ (4.8–10.2) in the TOT group (*p* = 0.42). The median necrotic tissue area was 3.4 cm^2^ (2.2–5.1) versus 4.1 cm^2^ (2.7–6.0), respectively (*p* = 0.37). There were no statistically significant differences in tissue composition at admission.

### 3.1. Primary Outcomes

The primary endpoint—total treatment duration—was markedly shorter in the intervention group, with a median of 10 weeks (IQR: 9–11) compared to 34 weeks (IQR: 23–37) in the TOT-only group (*p* = 0.008). Complete epithelialization was achieved in 67% of patients in the TOT + NPWT group versus 33% in the control group; this difference did not reach statistical significance (*p* = 0.18).

**Figure 1:**
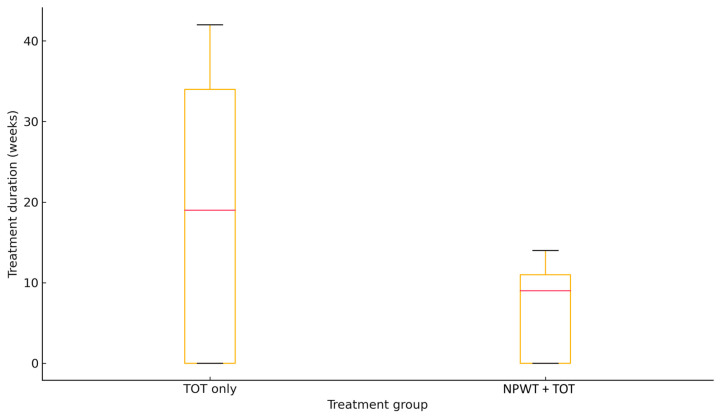
Treatment duration in the control and intervention group.

No treatment-related adverse events or complications occurred during the study period. Outcomes related to healing time and closure rate are summarized in Table 2 and Figure 1.

### 3.2. Wound Morphology and Thermographic Parameters

No statistically significant differences were observed between groups for the wound surface area, granulation tissue, or necrotic tissue on days 4 or day 7 (*p* > 0.05 for all comparisons). Similarly, no group-level differences were detected in wound bed or peripheral temperatures during the first week of treatment (*p* > 0.05). Median values and interquartile ranges for all wound morphometric parameters across the study days are presented in Table 3.

### 3.3. Ulcer Duration and Wound Characteristics

**Figure 2:**
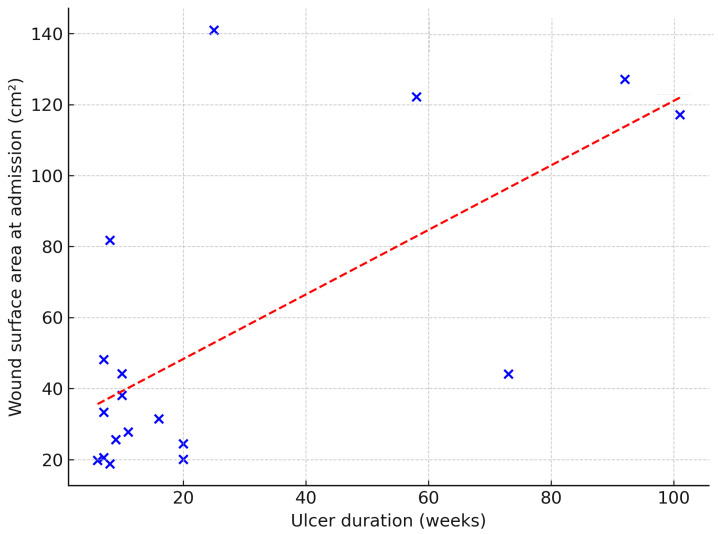
Relationship between ulcer duration (weeks) and wound surface area at admission (cm^2^). Dashed line indicates linear regression fit (β = 0.91; *p* = 0.003).

Ulcer duration showed no significant association with treatment duration or wound closure outcome (odds ratio = –0.06; *p* = 0.15). However, ulcer duration was significantly correlated with a larger wound surface area at baseline (β = 0.91 cm^2^ per week; *p* = 0.003) (Figure 2), as well as greater granulation (β = 0.51 cm^2^; *p* = 0.012) and necrotic tissue area (β = 0.45 cm^2^; *p* = 0.016).

### 3.4. Laboratory Biomarkers and Prognostic Associations

**Figure 3:**
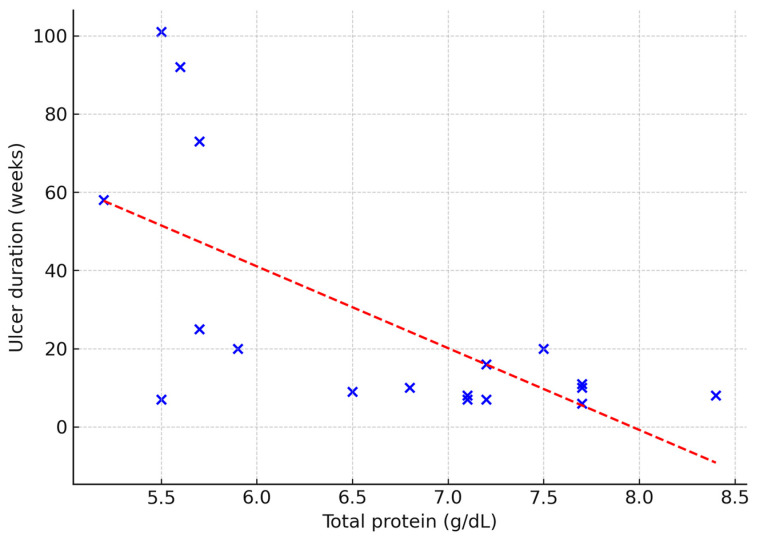
Relationship between total protein concentration and ulcer duration in weeks. Dashed line indicates linear regression fit (Spearman’s ρ = −0.53; *p* = 0.02).

**Figure 4:**
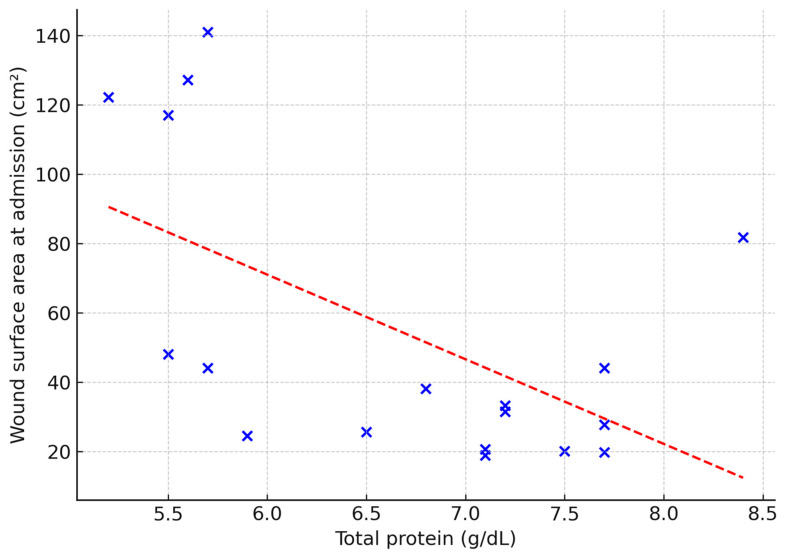
Relationship between total protein concentration and wound surface area at admission (cm^2^). Dashed line indicates linear regression fit (Spearman’s ρ = −0.50; *p* = 0.03).

**Figure 5:**
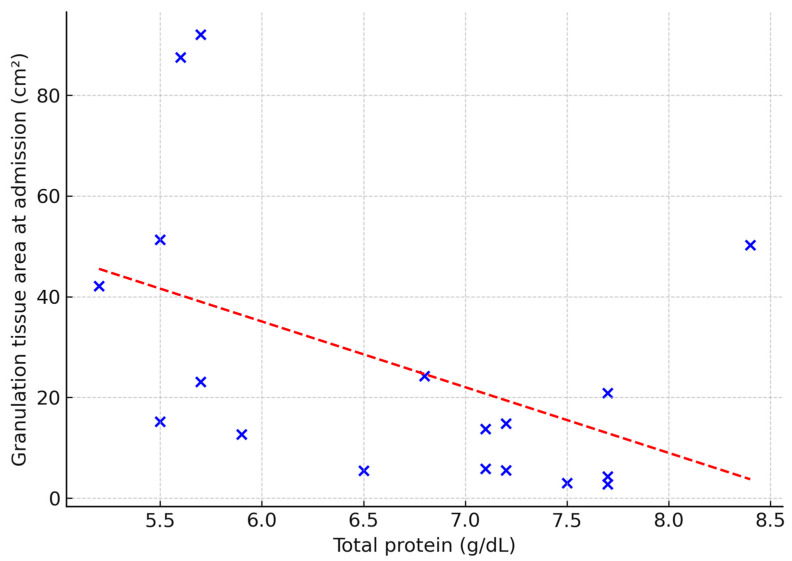
Relationship between total protein concentration and granulation tissue area at admission (cm^2^). Dashed line indicates linear regression fit (Spearman’s ρ = −0.53; *p* = 0.02).

The predictive value of selected laboratory parameters in chronic wound treatment was also explored. Serum total protein correlated negatively with ulcer duration (ρ = 0.53; *p* = 0.02), wound area (ρ = −0.50; *p* = 0.03) and baseline granulation tissue (ρ = –0.53; *p* = 0.02) (Figure 3, Figure 4 and Figure 5). No other laboratory markers, including CRP, NLR, MLR, or PLR, were significantly associated with these parameters (all *p* > 0.05).

### 3.5. Thermography and Inflammatory Ratio

**Figure 6:**
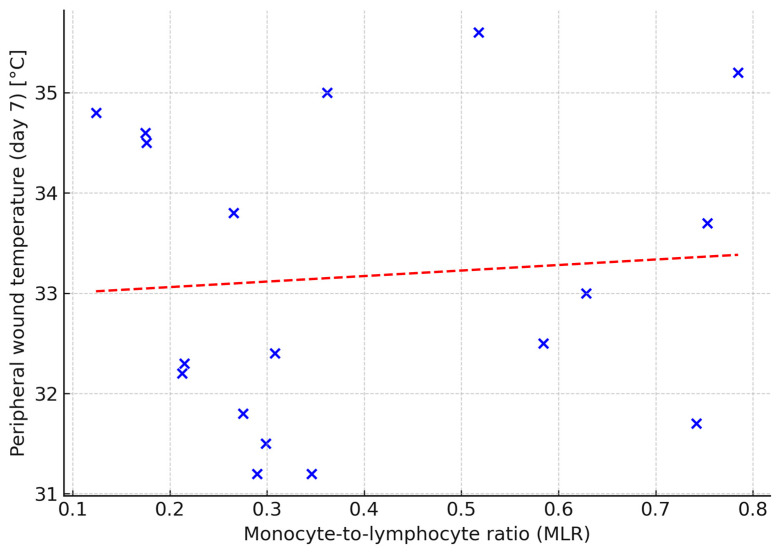
Relationship between monocyte-to-lymphocyte ratio (MLR) and peripheral wound temperature (1–2 cm from wound edge) on day 7 in control group. Dashed line indicates linear regression fit (Spearman’s ρ = 0.74; *p* = 0.02).

Thermal imaging revealed one statistically significant correlation: in the TOT-only group, the monocyte-to-lymphocyte ratio (MLR) on day 0 was positively correlated with peripheral wound temperature on day 7 (ρ = 0.74; *p* = 0.02) (Figure 6). No significant correlations were identified in the intervention group.

## 4. Discussion

The present pilot study demonstrates that adjunctive negative-pressure wound therapy (NPWT) administered after a short course of topical oxygen therapy (TOT) is associated with a clinically relevant reduction in overall healing time compared with TOT alone. Although the higher proportion of complete closures in our intervention arm did not achieve statistical significance, this numerical trend is consistent with findings from a recent randomized trial in advanced pressure ulcers, in which adjunctive topical oxygen shortened time-to-closure by approximately one month; a 2024 meta-analysis indicated a nearly two-fold increase in the likelihood of closure when oxygen delivery was combined with NPWT [9,10].

A significant association was also observed between ulcer duration and wound surface area, reinforcing the importance of early intervention in chronic wound care. These findings are consistent with prior studies indicating that prolonged inflammation in chronic wounds leads to progressive tissue damage and delayed healing [14,15].

Our data also highlight the potential prognostic value of serum total protein levels. The observed negative correlations between total protein and wound severity indicators (such as ulcer duration, wound area, and granulation tissue size) suggest that nutritional status may play an important role in wound healing, aligning with previous evidence linking protein-energy malnutrition to delayed epithelialization and higher infection risk [13]. Given its low cost and widespread availability, total protein could serve as a pragmatic triage marker, prompting early dietetic referral and targeted supplementation.

Inflammatory markers—including NLR, MLR, PLR, and CRP—have been proposed as indicators of systemic inflammation and tissue-repair capacity in chronic wounds; however, most published work has focused on their ability to stratify amputation risk in diabetic-foot syndrome rather than predicting healing kinetics per se [11,12,18]. In our study, only one statistically significant correlation was found: a strong positive relationship between MLR and peripheral wound temperature in the TOT-only group. This may reflect the interplay between systemic immune activation and local perfusion or inflammation. The absence of such correlations in the intervention group may suggest that NPWT modulates local tissue response, though the small sample size limits definitive conclusions.

Thermographic assessment of wound healing offers a promising non-invasive method to monitor local physiological changes [15]. Early wound-morphometry and thermography (day 7) did not differ between groups, indicating that macroscopic parameters measured within the first treatment week are insufficient to predict the longer-term divergence in healing trajectories. Animal data suggest that oxygen supplementation exerts its principal influence during the proliferative phase (post day 7), when collagen synthesis and neovascularization are at their maximum [19].

This study has several strengths, including prospective data collection, standardized infrared thermography, objective 3D wound assessment, and consistent care delivered by a dedicated wound-care team. However, important limitations must be acknowledged: a small sample size, lack of blinding, open allocation without concealment, and an exploratory statistical approach without adjustment for multiplicity. Nutritional status was assessed indirectly using serum total protein, without a comprehensive evaluation. Furthermore, the absence of standardized photographic documentation and microbial biofilm analysis limits the interpretation of wound evolution and infection dynamics. Thermographic monitoring was confined to the first treatment week, precluding the evaluation of delayed perfusion responses. Accordingly, our findings should be regarded as preliminary and hypothesis-generating.

In summary, the combination of TOT and NPWT was associated with a significantly shorter healing time compared to TOT alone. Serum total protein showed promise as a pragmatic biomarker of wound severity, while systemic inflammatory ratios were less informative.

### Future Directions

Building on these exploratory results, future research should pursue larger, randomized trials with longer thermographic follow-up and standardized photographic documentation. Integrating nutritional assessment, microbial analysis, and real-time monitoring of perfusion changes may enhance mechanistic understanding. Importantly, the use of prospective validation of serum protein and thermal signatures as predictive biomarkers could support the development of personalized, biomarker-guided wound care algorithms.

## 5. Conclusions

This pilot study suggests that combining topical oxygen therapy with negative-pressure wound therapy is feasible, safe, and potentially more effective than oxygen therapy alone at reducing healing time for chronic wounds. The observed correlation between ulcer duration and wound area highlights the importance of early intervention. Serum total protein may serve as a low-cost prognostic biomarker, although further validation is needed. Given the small sample size and non-randomized design of this study, these findings should be interpreted with caution.

Larger, rigorously designed studies are warranted to confirm therapeutic efficacy and to evaluate the clinical utility of integrating biomarkers—such as serum protein and thermographic data—into tailored treatment strategies for chronic wound patients.

## Figures and Tables

**Table 1:** Baseline characteristics in the study population.

Characteristic	TOT + NPWT (*n* = 9)	TOT Only (*n* = 9)	*p*-Value *
Age (years)	64 (60–76)	86 (70–86)	0.11
Female sex, *n* (%)	5 (56%)	5 (56%)	1.00
Ulcer duration (weeks)	16 (9–20)	10 (8–73)	0.96
Baseline wound area (cm^2^)	27.8 (24.5–48.1)	44.1 (33.3–81.8)	0.51
Serum total protein	7.0 (5.7–7.2)	6.9 (5.9–7.4)	0.83

* Mann–Whitney U test for continuous variables; Fisher’s exact test for categorical variables. Values are shown as the median (interquartile range).

**Table 2:** Summary of treatment duration and complete wound closure rates in NPWT + TOT and TOT-only groups.

Treatment Group	Treatment Duration (Median, IQR ^1^) (Weeks)	Complete Wound Closure *n* (%)
NPWT + TOT	10 (9–11)	6 (66.7%)
TOT only	34 (23–37)	3 (33.3%)

^1^ IQR: interquartile range.

**Table 3:** Temporal changes in wound morphology.

Day	Wound Area (TOT + NPWT), cm^2^	Wound Area (TOT Only), cm^2^	Granulation Tissue (TOT + NPWT), cm^2^	Granulation Tissue (TOT only), cm^2^	Necrotic Tissue (TOT + NPWT), cm^2^	Necrotic Tissue (TOT Only), cm^2^
Day 0	27.8 (24.5–48.1)	44.1 (33.3–81.8)	9.2 (6.1–14.5)	6.3 (4.8–10.2)	3.4 (2.2–5.1)	4.1 (2.7–6.0)
Day 4	25.2 (22.3–41.7)	39.7 (30.2–72.0)	12.1 (9.7–18.3)	7.1 (5.4–12.5)	2.0 (1.1–3.4)	3.6 (2.1–5.4)
Day 7	22.2 (17.4–42.9)	34.9 (26.3–72.3)	15.5 (11.9–21.1)	8.2 (6.2–13.8)	1.2 (0.7–2.1)	2.9 (1.8–4.7)

Data are presented as median (interquartile range).

## Data Availability

The data are not publicly available due to ethical and privacy restrictions imposed by the institutional review board. An anonymized dataset may be made available by the corresponding author upon reasonable request and pending ethics approval.

## Abbreviations

The following abbreviations are used in this manuscript:

TOT	Topical Oxygen Therapy
NPWT	Negative-Pressure Wound Therapy
NLR	Neutrophil-to-Lymphocyte Ratio
MLR	Monocyte-to-Lymphocyte Ratio
PLR	Platelet-to-Lymphocyte Ratio
CRP	C-Reactive Protein
